# Distribution of proviral DNA of bovine leukaemia virus in blood and different tissues in asymptomatically infected cattle

**DOI:** 10.2478/jvetres-2025-0063

**Published:** 2025-11-07

**Authors:** Anna Ryło, Marzena Rola-Łuszczak, Jacek Michał Kuźmak

**Affiliations:** Department of Virology and Viral Animal Diseases, National Veterinary Research Institute, 24-100 Puławy, Poland

**Keywords:** bovine leukaemia virus, molecular detection of bovine lymphoma, qPCR, solid tissue amplification, viral genome integration

## Abstract

**Introduction:**

Bovine leukaemia virus (BLV) is the aetiological agent of enzootic bovine leukosis. The aim of the study was to ascertain the ability of qPCR to detect proviral BLV DNA in various tissues from slaughtered cattle when BLV was suspected but serological testing was not possible.

**Material and Methods:**

Three types of tissues were collected during sanitary slaughtering of 22 cattle naturally infected with BLV: spleen, lymph node and muscle. The proviral load (PVL) was estimated in this tissue by a real-time quantitative PCR (qPCR) for BLV based on the *pol* gene. To measure provirus copy number per 10^6^ cells, the bovine histone H3 family 3A gene was also amplified by qPCR.

**Results:**

The PVL was the highest in the spleen and ranged there from 1 to 59,188 copies/10^6^ cells, with four cases in which no proviral DNA was detected. In the lymph nodes the PVL ranged from 2 to 6,888 copies/10^6^ cells, with seven cases in which no copies were detected. The lowest PVL was recorded in DNA from muscle samples and ranged from 1 to 119 copies/10^6^ cells; no BLV was detected in 6 out of 22 samples.

**Conclusion:**

The BLV qPCR is a suitable tool for the detection of proviral BLV DNA in various tissues when infection is suspected and no blood or other fluids are available for serological examination.

## Introduction

Bovine leukaemia virus (BLV) is the aetiological agent of enzootic bovine leukosis (EBL). Most BLV-infected cattle remain asymptomatic, but approximately 30% develop persistent lymphocytosis. Among infected animals, 1–5% develop malignant tumours (lymphosarcoma), these predominantly being adult cattle older than 3–5 years (19). Lymphosarcoma involves enlarged lymph nodes, in which normal histological structure is lost. Lesions in BLV infections most often affect the spleen, liver, kidneys and ureters (2).

While not touching most European countries, BLV infection appears to be widespread globally. The prevalence of the virus varies from country to country, ranging from 30% to 80%, and is generally higher in dairy cattle (12). In BLV-infected cattle, seroconversion occurs from two weeks to three months after infection, after which time detection of specific antibodies in serum samples by ELISA or agar gel immunodiffusion tests is possible; these are the methods recommended by the World Organisation for Animal Health (WOAH) for serological diagnosis of EBL. A PCR can be used as an adjunct to serology for confirmatory testing. However, this method is also recommended for tissue examination from suspected cases collected at abattoirs during post-mortem carcass examination (22).

Poland has been recognised as officially EBL free since May 2017. As the National Reference Laboratory for enzootic bovine leukosis, the National Veterinary Research Institute fills a crucial role in the national surveillance system. It is the provider of confirmation of suspected BLV cases identified during ongoing monitoring surveys in the country. The Institute has developed a qPCR method for the BLV *pol* gene which has proven to be very sensitive and has allowed the disease status of animals to be elucidated by direct detection of proviral BLV DNA in peripheral blood leucocytes (PBLs) or lymph nodes of animals suspected of being infected (18). This real-time quantitative PCR method (qPCR) is among the validated tests in the WOAH Manual of Diagnostic Tests and Vaccines for Terrestrial Animals, and is successfully used in several international laboratories performing EBL diagnosis (17).

The testing of slaughtered cattle is a tool to reduce the risks to the safety and health of consumers, is carried out systematically, and serves to deem carcasses showing neoplastic lesions or tumours unfit for human consumption (10). Beside lymphosarcoma related to EBL, other forms of lymphomas with different aetiology called sporadic bovine leukosis can be found in slaughtered cattle. These include calf, thymic and skin forms. Histological examination can support the diagnosis of malignant lymphomas, but is not able to distinguish between sporadic lymphomas and those induced by BLV if no blood or other fluids are available for serological examination. In such cases a PCR directly detecting proviral DNA in selected tissues from carcasses is the method of choice (8). Submission of all lymphomas for laboratory testing for the presence of BLV infection is an additional component of the surveillance system which is crucial for maintaining a country’s freedom from BLV. This testing is obligatory because of a provision of EU animal law that EBL-free status may be granted to a Member State or zone if at least 99.8% of cattle holdings are EBL free, all animals over two years of age that are slaughtered undergo post-mortem examination, and tumour samples are tested in a laboratory to rule out EBL infection (4).

The present study aimed to determine whether a developed qPCR could detect proviral BLV DNA in various tissues as effectively as in PBLs of naturally infected cattle. The selected method combined the qPCR with amplification of the *H3F3A* reference gene, which allows the number of copies of proviral BLV DNA in a given number of cells to be accurately assessed (16). In addition, taking advantage of the high sensitivity of qPCR, the optimal tissue was determined for BLV testing during routine carcass examination in cases when the presence of neoplastic lesions or tumours is suspected and serological testing is not possible.

## Material and Methods

Blood samples were collected by veterinary inspectors working on the EBL monitoring programme in Poland in 2016–2017, and were tested by regional veterinary laboratories. Blood from serologically positive cattle was then submitted to the National Reference Laboratory to be confirmed by ELISA (Leukosis Ab Blocking test, IDEXX Laboratories, Montpellier, France) and a qPCR using genomic DNA extracted from PBLs, as was described previously (18). Twenty-two cattle originating from five different voivodeships and aged from 1.5 to 14 years were slaughtered because they were positive for BLV by ELISA and qPCR, and fragments of the spleen; the mesenteric, pre-scapular and mediastinal lymph nodes; and the diaphragm, shoulder and chuck roast muscles were collected by veterinary inspectors. Hearts, lungs, livers and kidneys were also collected, but not from all animals; therefore, these specimens were not used in this study.

Tissue samples were transported to the laboratory in separated plastic bags in frozen conditions and stored at –20°C until laboratory analysis. Tissues were then fragmented with a sterile scalpel blade, and approximately 25 mg of each was used for the isolation of genomic DNA with a NucleoSpin Tissue kit (Macherey-Nagel, Düren, Germany) according to the manufacturer’s instructions. The DNA concentration was estimated by spectrophotometry using a NanoPhotometer (Implen, Munich, Germany). To estimate BLV proviral load (PVL), 500 ng of genomic DNA was amplified by BLV qPCR (18).

The bovine histone H3 family 3A (*H3F3A*) gene was amplified by qPCR to calculate the frequency of infected cells per 10^6^ cells (16). Prior to this, the usefulness of this gene and *RPL15* (ribosomal protein L15) was ascertained for determining the number of cells taken for amplification in 500 ng of genomic DNA isolated from different cattle organs. For this purpose a comparative study was conducted on a panel of 20 DNA samples representing four types of tissue: leukocytes, muscles, spleen and lymph nodes. The amplification results obtained for both reference genes were compared, and it was found that the *H3F3A* gene yielded stable results as a reference gene. The variability (coefficient of variation) for this gene ranged from 1 to 4% and the difference in threshold cycle (Ct) was less than 2 for each tissue sample, which is acceptable for a given number of copies. The results for the *RPL15* gene did not meet these criteria.

The correlation and the slope were calculated by use of Microsoft Office Excel software. The normality of the number of proviral BLV DNA copies per 10^6^ cells in each tissue was evaluated by the Shapiro–Wilk test, and the Kruskal–Wallis test was used to identify differences between groups. Statistical significance was defined as a P-value of less than 0.05.

## Results

To estimate the distribution of BLV proviral load in different tissues, 22 cattle positive for BLV infection were selected and slaughtered. Veterinary inspectors did not identify any pathological symptoms or lesions in the carcasses or organs in slaughtered animals at the time of sample collection. In total, 66 genomic DNA samples coming from tissues of 22 cattle were analysed. Mesenteric, pre-scapular and mediastinal lymph nodes were provided, but in many cases the samples were not properly identified, and consequently these samples needed all to be categorised simply as lymph nodes.

**Table 1.** Bovine leukaemia virus (BLV) proviral load in peripheral blood leukocytes (PBLs) and organs of naturally infected cattle

Carcass No.	BLV proviral load – number of copies per 10^6^ cells			
	PBLs	muscles	spleens	lymph nodes
1	85,803	119^*^	59,188	881
2	57,284	114^*^	58,883	6,888
3	54,556	14^**^	9,559	1,419
4	38,228	51^*^	645	278
5	14,895	19^*^	3	287
6	11,019	13^*^	3,215	54
7	8,603	20^*^	314	14
8	7,323	35^*^	3,231	85
9	5,330	4^*^	2,295	24
10	2,211	0^*^	45	111
11	269	31^*^	37	96
12	187	2^*^	1	0
13	182	0^*^	0	0
14	25	0^*^	48	6
15	16	0^*^	17	2
16	11	8^***^	0	8
17	11	0^*^	2	0
18	4	0^*^	4,137	0
19	3	4^***^	8	0
20	3	4^*^	1	0
21	3	1^*^	0	4
22	1	4^*^	0	0

1 Anatomical location of tested muscles:

1 * – diaphragm;

1 ** – shoulder;

1 *** – chuck roast

The BLV proviral load in PBLs ranged from 1–85,803 copies/10^6^ cells (median value 128.0). The PVL not in PBLs was the highest in the spleen, and ranged from 1–59,188 copies/10^6^ cells (median value 41.0), and the mean was 6,438 copies/10^6^ cells. Four cases were noted in which no proviral DNA was detected (18%). In the lymph nodes, the PVL ranged from 2–6,888 copies/10^6^ cells (median value 11.0), and the mean was 462 copies/10^6^. Seven samples had no copies detected (32%). The lowest PVL was recorded in DNA from muscle samples and ranged from 1–119 copies/10^6^ cells (median value 4.0), and BLV was absent from 6 out of 22 samples (27%). The mean PVL in muscle samples was 20 copies/10^6^ cells, which indicated that this tissue contained the lowest concentration of BLV among the samples analysed in this study. The Kruskal–Wallis test showed that differences in BLV proviral DNA levels were statistically significant between groups of tissues (P-value < 0.05).

**Fig. 1.**
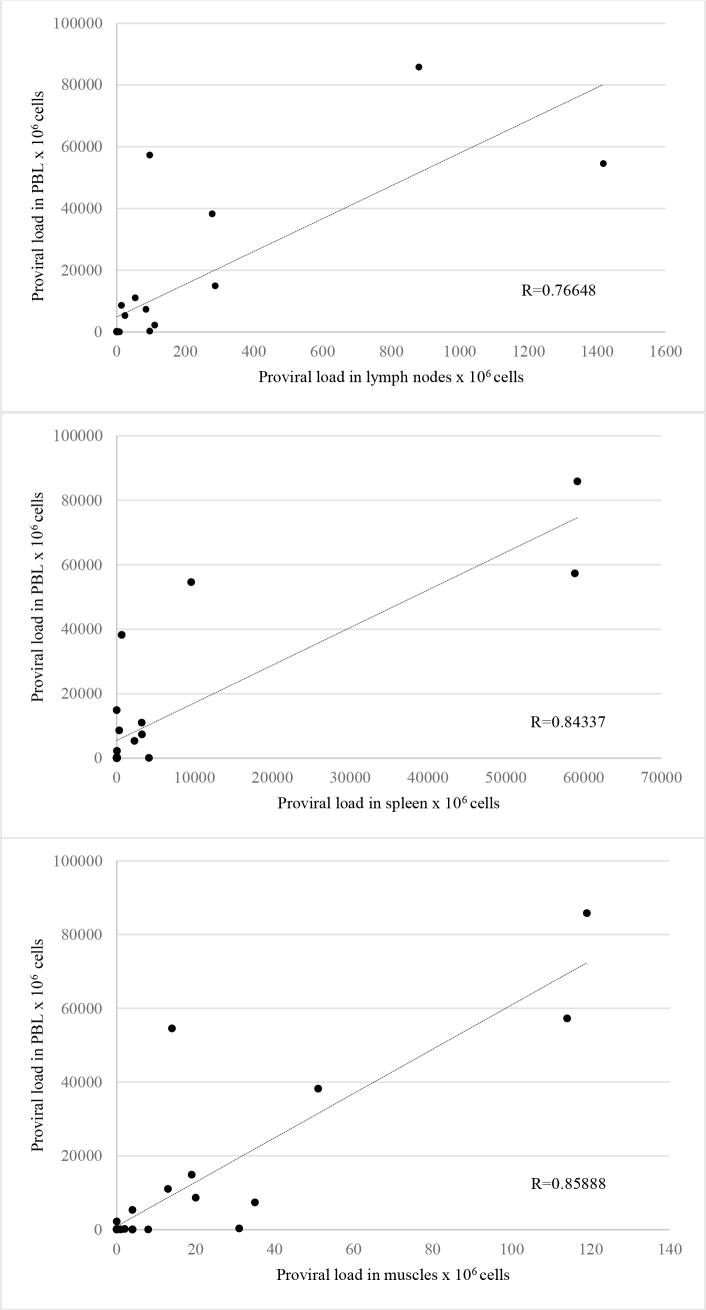
Correlations between peripheral blood leukocyte (PBL) bovine leukaemia virus proviral load (PVL) and the PVL in the examined lymph nodes, muscles and spleens. The BLV PVL is expressed as number of copies of proviral DNA per 10^6^ cells

Subsequently the correlations between the level of BLV proviral DNA in PBLs and its levels in muscles, spleens and lymph nodes were calculated (Fig.1). In all examined organs, the correlation coefficient was within the range 0.7 ≤ |R| < 0.9, which indicated strong correlation. The highest correlation was noted between the level of proviral DNA in PBLs and its level in muscle (R = 0.85888). The coefficient between PBL proviral DNA and spleen proviral DNA was only marginally lower (R = 0.84337), and between PBLs and lymph nodes the correlation was lower by a larger margin (R = 0.76648).

## Discussion

In this study we demonstrated the suitability of the qPCR method to perform quantitative analysis of BLV proviral load in DNA isolated from spleens, lymph nodes and muscles of BLV-infected cattle. Our intention was to create a new diagnostic platform for detecting BLV infections during routine carcass examination when no blood or other fluids can be obtained for serological testing. Selection of tissues designated for our analysis was informed by the known tropism of BLV noted in previous reports (7, 11, 14, 20). The qPCR designed for this research was successfully applied in the detection of proviral BLV DNA in genomic DNA extracted from all tested tissues, even at low copy numbers of proviral DNA (1–20 copies per 10^6^ cells). For a qPCR with this purpose, reaction product quality is as important as the reaction’s useability with low-burden tissue. The genomic DNA isolated from each type of tissue was of adequate quality for real-time PCR amplification, as indicated by the stable amplification of the *H3F3A* reference gene, with the average Ct values of 21.2 (±1.07), 20.3 (±0.58) and 20.6 (±0.51) in spleen, lymph node and muscle samples, respectively.

Twenty two cattle were selected with different proviral BLV DNA levels in PBLs. The highest number of provirus copies reached 85,803 per 10^6^ cells, and the lowest was 1 copy per 10^6^ cells. However, 51% of PBL samples yielded provirus concentrations in the range below 1,000 copies per 10^6^ cells, as might be expected for asymptomatic cattle. Interestingly, with regard to the non-blood tissues examined, the most common result for copy concentration was also up to 1,000 copies per 10^6^ cells. A 67% proportion of spleen tissue samples, a 91% proportion of lymph node samples and 100% of muscle samples contained no more than 1,000 copies per 10^6^ cells. The tissue virus burden results showing the spleen and lymph nodes more highly infiltrated than muscle are in line with those of the study of Kohara *et al*. (9). They also observed high concentrations of proviral DNA in the spleen and lymph nodes of experimentally infected cattle. Regarding muscle samples, the level of provirus was found in this tissue as the lowest, from 1 to 119 copies/10^6^ cells, and BLV genome portions were detected in 73% of samples (16/22). The issue of the presence of BLV proviral DNA in raw beef was investigated by Olaya-Galán *et al*. (15). In that study, as in our research, the proviral load was low in the meat samples, and most positive results were only detected by nested PCR. Interestingly, we noted high concordance of positive results between lymph node and muscle samples (15/22 and 16/22, respectively), despite the far higher PVL concentration in lymph nodes (median 11.0) than in muscle (median 4.0). However, the samples with a high PVL (sampled carcasses 1–11) were the only ones in accordance across tissue types, while samples with a relatively low PVL (carcasses 12–22) differed. These results clearly indicate the usefulness of diagnostic testing of DNA isolated from solid tissue when whole blood is unavailable. Our results revealed that proviral BLV DNA in PBLs positively correlated with distribution of provirus in spleen, muscle and lymph node samples in naturally infected cattle. This finding is consistent with a similar trend observed in experimentally infected cattle (9). Nevertheless, in some animals, proviral DNA was not detected in certain tissues, despite ongoing infection. Such situations can be explained by the low PVL in these tissues, which may be related to the carcasses having been those of cattle in the initial phase of infection, when PBLs are the main reservoir of the virus.

Based on published data from sanitary and veterinary inspections from Poland, single tumours in slaughtered cattle and a low number of lymphoma lesions were found in 2003 and 2016, respectively (6, 13). Also, in the last 10 years, there has only been need for tumour samples (from six slaughtered cattle) to be submitted to the National Reference Laboratory for confirmatory diagnosis. These samples were taken from carcasses showing lymph node enlargement and lesions suggesting a neoplastic nature, but they were negative for BVL by qPCR. Similarly, in the EU in 2009–2015, only 35 lymphoma cases were reported from all Member States. It seems that the incidence of lymphomas caused by BLV infection is probably underestimated. This would be principally because not all cases of lymphoma are reported: some affected animals die on farms, and stock is partially withdrawn from slaughterhouses, and in these circumstances no BLV cases are recorded even if they were factual. This is a problem at the European level, as fewer suspected cases of lymphomas are reported and investigated than the real situation merits (5). Even if this data indicates a relatively low incidence of tumorous lesions in carcasses of slaughtered cows, research aimed at identifying these changes, primarily those caused by BLV, should be conducted as part of a surveillance programme.

Our studies have shown that qPCR may be useful in this regard, as it allows for rapid and sensitive detection of BLV proviral DNA in post-mortem testing in cases of suspected tumorous lesions. This qPCR testing possibility may also be welcome when acknowledging uncooked or partially cooked meat and unpasteurised milk derived from BLV-infected cattle as potential zoonotic infection sources in humans, as has been suggested (3). A hypothetical scheme of BLV transmission to humans was presented by Blanco *et al*. (1). In this theoretical transmission route, proviral BLV DNA is distributed from the milk and meat of BLV-infected cattle in the following manner: after being consumed with milk or meat, the virus can pass into blood and circulate in the bloodstream all over the body. Indeed, Corredor-Figuerosa *et al*. (3) found a correlation between higher milk consumption and the risk of BLV infection, but this relationship was not confirmed for meat ingestion.

## Conclusion

The selected qPCR method was suitable to perform quantitative analysis of proviral load in different tissues of BLV-infected cattle. Proviral load in blood was generally well correlated with copy numbers found in other tissues, spleen burden being the highest among the samples tested (spleen, muscle and lymph nodes). This may be a direct indication to perform qPCR testing of tissues during routine carcass examination when serum samples are not available but suspected lesions like tumours or lymphomas are found. The developed BLV qPCR is a suitable tool to detect proviral BLV DNA in various lesion-bearing tissues when whole blood is not available for serological and molecular testing.
